# Identification of the Microbiota in Carious Dentin Lesions Using 16S rRNA Gene Sequencing

**DOI:** 10.1371/journal.pone.0103712

**Published:** 2014-08-01

**Authors:** Junko Obata, Toru Takeshita, Yukie Shibata, Wataru Yamanaka, Masako Unemori, Akifumi Akamine, Yoshihisa Yamashita

**Affiliations:** 1 Section of Preventive and Public Health Dentistry, Division of Oral Health, Growth and Development, Kyushu University Faculty of Dental Science, Fukuoka, Japan; 2 Department of Endodontology and Operative Dentistry, Division of Oral Rehabilitation, Kyushu University Faculty of Dental Science, Fukuoka, Japan; Columbia University, United States of America

## Abstract

While mutans streptococci have long been assumed to be the specific pathogen responsible for human dental caries, the concept of a complex dental caries-associated microbiota has received significant attention in recent years. Molecular analyses revealed the complexity of the microbiota with the predominance of *Lactobacillus* and *Prevotella* in carious dentine lesions. However, characterization of the dentin caries-associated microbiota has not been extensively explored in different ethnicities and races. In the present study, the bacterial communities in the carious dentin of Japanese subjects were analyzed comprehensively with molecular approaches using the16S rRNA gene. Carious dentin lesion samples were collected from 32 subjects aged 4–76 years, and the 16S rRNA genes, amplified from the extracted DNA with universal primers, were sequenced with a pyrosequencer. The bacterial composition was classified into clusters I, II, and III according to the relative abundance (high, middle, low) of *Lactobacillus*. The bacterial composition in cluster II was composed of relatively high proportions of *Olsenella* and *Propionibacterium* or subdominated by heterogeneous genera. The bacterial communities in cluster III were characterized by the predominance of *Atopobium*, *Prevotella*, or *Propionibacterium* with *Streptococcus* or *Actinomyces*. Some samples in clusters II and III, mainly related to *Atopobium* and *Propionibacterium*, were novel combinations of microbiota in carious dentin lesions and may be characteristic of the Japanese population. Clone library analysis revealed that *Atopobium* sp. HOT-416 and *P. acidifaciens* were specific species associated with dentinal caries among these genera in a Japanese population. We summarized the bacterial composition of dentinal carious lesions in a Japanese population using next-generation sequencing and found typical Japanese types with *Atopobium* or *Propionibacterium* predominating.

## Introduction

Dental caries is the most prevalent bacteria-related disease worldwide; approximately 2.43 billion people, 36% of the world’s population, have carious lesions in permanent teeth [1]. Although reductions in dental caries prevalence have been reported in many developed countries due to preventative measures, including fluoride application, sealants, improvements in diet, oral health education, and oral healthcare, recent epidemiological data have shown a reversing situation and an alarming increase in the global prevalence of dental caries during the past decade [2].

Mutans streptococci had long been assumed to be the specific pathogen responsible for human dental caries among the hundreds of oral bacterial species. Their abilities to form a rigid biofilm on smooth tooth surfaces and to continuously produce acid, even under acidic conditions, are key properties for enamel demineralization, an early step in cariogenesis [3]. However, no association between high counts of mutans streptococci and caries severity has been reported [4], [5]. Other evidence that does not support the possible role of mutans streptococci in dental caries includes similar mutans streptococcus counts among children from Africa, Europe, and North America with different caries rates and radical differences in racial, cultural, and social aspects [6]. These facts indicate that variation in the caries prevalence pattern in those dissimilar populations is not attributable to the level of *Streptococcus mutans* infection. On the other hand, the oral plaque biofilm is composed of a large number of bacterial species, and the dental caries-associated microbiota is highly complex. Therefore, a comprehensive analysis is needed to determine the actual cariogenicity of the oral microbiota.

Dentinal caries provokes serious problems, including toothache and pulp infection. Classical cultivation approaches have resulted in the frequent isolation of *Lactobacillus*, *Actinomyces*, and *Streptococcus* species with anaerobic or facultative Gram-positive rods from carious dentin [3], [7]; mutans streptococci were not always detected as major members of the bacterial communities in dentinal caries. Furthermore, strictly anaerobic cultivation conditions have predominantly resulted in the isolation of *Propionibacterium*, *Eubacterium*, *Arachnia*, *Lactobacillus*, *Bifidobacterium*, and *Actinomyces* species from lesions [8], suggesting that the bacterial compositions in enamel and dentinal caries differ fundamentally. However, the pathological process by which enamel caries converts to dentinal caries remains to be determined. Recent molecular analyses of the microbial diversity in dentinal caries revealed that *Lactobacillus* is most frequently detected in many dentinal caries lesions, with some variation in the amounts of several accompanying genera [9], [10]. In particular, *Prevotella* was found to be a second predominant genus in dentinal caries [10]–[12]. However, the heterogeneous composition of the microbiota in dentinal caries has not been explored extensively in different ethnicities and races.

Although dental caries exhibits aspects of an infectious disease caused by oral bacteria, as described above, its etiology is multifactorial [13]. In particular, diet is a crucial factor in caries incidence. Dietary conditions vary among ethnicities, and dietary culture cannot be excluded when caries etiology in a specific ethnicity is considered. The staple food in Japan is steamed rice, which is rich in carbohydrates and sticks to tooth surfaces. The Japanese dietary culture can be distinguished from those of other countries. Thus, the pathogens in dentinal caries in Japanese patients may differ from those reported in other countries. In addition, it was recently revealed that the oral microbiota is diverse among different geographical sites [14] and ethnicities [15], and these discrepancies might explain the diversity in dentin caries microbiota compositions among different ethnicities and races.

In the present study, bacteria occupying the front-most layers of dentinal caries were analyzed comprehensively using pyrosequencing of 16S rRNA amplicons and microbiota that might be critical in dentin destruction were analyzed qualitatively in a Japanese population.

## Materials and Methods

### Ethics statement

Written informed consent was obtained from all adult participants and parents of child participants. The ethics committee of Kyushu University approved this study and the procedure for obtaining informed consent.

### Study population

The study population comprised 32 patients (17 males and 15 females) aged 4–76 years. A total of 12 participants were patients at Kyushu University Hospital, and 20 were patients at the YA Dental Clinic in Yonago, Japan.

### Sample collection and DNA extraction

A carious tooth was selected from each subject and isolated with a rubber dam before sample collection. After the removal of dental plaque, enamel and a shallow layer of dentin were removed with a sterile diamond bur, and carious dentin was hand-excavated with sterile spoon excavators. Large carious dentin samples were divided according to depth, placed into two or three sterile microcentrifuge tubes, and analyzed separately. The samples were frozen at –30°C until analysis. Bacterial DNA extraction was performed as described previously [16].

### Pyrosequencing analysis

The 16S rRNA genes of each sample were amplified using the following primers: 338R with the 454 Life Sciences adaptor B sequence (5′-CCT ATC CCC TGT GTG CCT TGG CAG TCT CAG TGC TGC CTC CCG TAG GAG T-3′) and 8F with the 454 Life Sciences adaptor A and subject-specific six-base barcode sequences (5′-CCA TCT CAT CCC TGC GTG TCT CCG ACT CAG NNN NNN AGA GTT TGA TYM TGG CTC AG-3′). The PCR amplicons were gel-purified using a Wizard SV Gel and PCR Clean-Up System (Promega, Madison, WI) according to the manufacturer’s protocol. The DNA concentration and quality were assessed using a NanoDrop spectrophotometer (NanoDrop Technologies, Wilmington, DE), and equal amounts of DNA from the 44 samples were pooled. Pyrosequencing was conducted using a 454 Life Sciences Genome Sequencer FLX instrument (Roche, Basel, Switzerland).

### Data analysis and taxonomic assignment

Sequences of <240 bases or with average quality scores <25 were excluded from the analysis using a script written in PHP, and those that did not include the correct primer sequence, had a homopolymer run ≥7 nt, or contained ambiguous characters were subsequently removed using a script written in R. The remaining sequences were assigned to each subject by examining the six-base barcode sequence. Each sequence was compared using blastn function with the default parameters in BLAST 2.2.27+ (megablast) with 831 sequences in HOMD 16S rRNA RefSeq Version 13.2 which are the curated reference sequences of oral bacterial 16S rRNA gene deposited in the Human Oral Microbiome Database [17]. The sequences were assigned to the best BLAST hit with a 98.5% similarity value and minimum coverage of 97%.

After excluding assigned sequences, similar sequences were clustered into operational taxonomic units (OTUs) using the complete-linkage clustering tool of the RDP pyrosequencing pipeline [18] at a distance cut-off of 0.03, and representative sequences of each cluster were selected using the Dereplicate request function. The representative sequences from each OTU were then aligned using PyNAST [19] and the Greengenes database [20] with a minimum percentage identity of 75%. Chimeras were removed from the representative set on the basis of identification as chimeric using Chimera Slayer [21]. After chimera elimination, the taxonomy of the representative sequence of each OTU was determined using the RDP Classifier with a minimum support threshold of 60% and the RDP taxonomic nomenclature (at the genus level).

A relaxed neighbor-joining tree was built using FastTree [22]. To determine the degree of dissimilarity between pairs of bacterial communities, we used the UniFrac metric [23], calculated with Fast UniFrac [24]. UniFrac distances are based on the fraction of branch length shared between two communities within a phylogenetic tree constructed from all communities being compared. The similarity relationship, assessed using the weighted UniFrac metric, was represented in a principal coordinate analysis (PCoA) plot, drawn with R. Bacterial genera composition patterns of microbiota were classified by hierarchical cluster analysis using Euclidean distance and Ward’s method. The obtained sequence data were deposited in DDBJ Sequence Read Archive under accession number DRA002215.

### Cloning and sequence analysis

Seven out of 32 samples representing subjects who participated in this study were excluded from the cloning and sequence analysis due to an insufficient amount of sample after the barcoded pyrosequencing analysis. DNA fragments were amplified using unlabeled bacterial universal primers 8F (5′-AGA GTT TGA TYM TGG CTC AG-3′) and 1492R (5′-GGY TAC CTT GTT ACG ACT T-3′), and gel-purified using a Wizard SV Gel and PCR Clean-Up System (Promega). Purified amplicons were cloned into the vector pBluescript II SK (+) (Stratagene, La Jolla, CA). The nucleotide sequences of the inserts were determined using the M13 (-40) forward primer. The sequence data were extended to at least 500 base pairs from the forward primer. For each sequence, the nearest-neighbor species with >98% identity were first searched using BLAST using blastn function with the default parameters in BLAST 2.2.27+ (megablast) against the reference sequences in HOMD 16S rRNA RefSeq Version 13.2 [17]. Sequences with no hits were further compared against the database Nucleotide collection (nr/nt) of the National Center for Biotechnology Information (http://www.ncbi.nlm.nih.gov/BLAST/) using BLASTn optimized for megablast with default parameters.

### Statistical analysis

Student’s t-test for comparing UniFrac distance was performed using R software [25]. Statistical significance was set at *P*<0.05.

## Results

Carious dentin samples were collected from 32 decayed teeth of 32 patients visiting Kyushu University Hospital or YA Dental Clinic. Nine thick dentinal lesion samples were divided into two or three layers according to depth; thus, a total of 44 samples were analyzed. The patient’s general conditions and details of the dental caries regions are described in Table 1.

**Table 1:** General condition of the 32 patients and details of dental caries regions.

Sample ID	Gender	Age	Tooth position^a^	Filling	Sample collection^b^
I-1	Male	11	16	−	
I-2	Male	12	46	−	
I-3	Female	70	24	−	divided into 2 samples
I-4	Male	7	85	−	
I-5	Male	27	26	−	
I-6	Male	9	55	−	
I-7	Male	4	64	−	
I-8	Female	28	47	−	
I-9	Male	5	75	−	
II-1	Female	23	25	−	
II-2	Male	30	17	−	
II-3	Male	6	75	−	
II-4	Male	13	46	−	
II-5	Female	55	23	+	divided into 2 samples
II-6	Female	7	85	−	
II-7	Female	5	55	−	
II-8	Female	32	27	+	divided into 2 samples
II-9	Male	7	75	−	
II-10	Female	36	34	+	divided into 3 samples
II-11	Male	52	13	+	
II-12	Female	13	26	+	
III-1	Male	15	16	+	
III-2	Male	10	46	+	divided into 2 samples
III-3	Female	26	47	−	
III-4	Female	45	26	+	
III-5	Female	7	84	−	
III-6	Female	37	34	−	divided into 2 samples
III-7	Male	76	22	+	divided into 3 samples
III-8	Female	52	13	−	divided into 2 samples
III-9	Male	6	65	−	
III-10	Female	38	45	−	
III-11	Male	48	24	−	divided into 3 samples

a The tooth position was described based on two-digit tooth numbering system defined by World Dental Federation (FDI).

b Large carious dentin samples were divided according to depth, placed into two or three different tubes.

Whole genomic DNA was extracted from each sample and the variable regions (containing the V1–V2 region) of the bacterial 16S rRNA gene were PCR-amplified using universal primers with sample-specific tag sequences. Using the Roche 454 FLX instrument, we determined 102,546 bacterial 16S rRNA gene sequences (containing the V1–V2 region), of which 92,520 passed quality control tests (2,102±503 reads per sample). The 74,314 of these sequences were assigned to 297 oral taxons (346 sequences) in the oral bacterial 16S rRNA gene sequences deposited in HOMD [17] with a high similarity (≥98.5%). The unassigned 18,206 sequences were classified into 1,858 OTUs using a cut-off distance of 0.03, whereas 1,128 of these OTUs consisted of only one or two reads.

**Figure 1:**
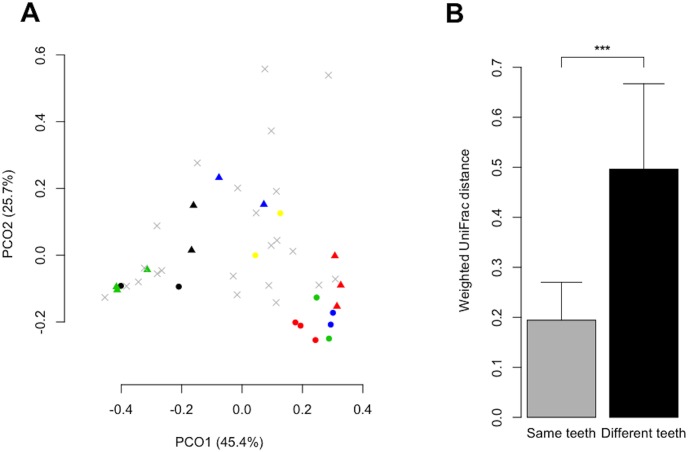
UniFrac analysis of the microbiota in carious dentin lesions. A. Principal coordinate analysis (PCoA) diagram based on weighted UniFrac distance showing the similarity relationships among the 44 carious dentin samples collected from 32 subjects. These two components explained 71.1% of the variance. Multiple samples collected from the same caries region are plotted in the same shape and color. Single samples are plotted as a gray cross (×). B. Weighted UniFrac distances between samples collected from the same caries region (the same teeth) and those between samples collected from the deepest layer of dental caries regions of different subjects (different teeth). ***P<0.001, Student’s t-test.

The overall bacterial community composition was compared using UniFrac, a phylogeny-based distance metric ranging from 0 (identical bacterial communities) to 1 (completely different communities). Samples collected from the same teeth were located close to each other on a PCoA plot based on weighted UniFrac values (Figure 1A). Variations in bacterial community composition between samples collected at different depths from the same caries region were significantly smaller than inter-individual differences (Student’s *t*-test, *P*<0.001; Figure 1B).

**Figure 2:**
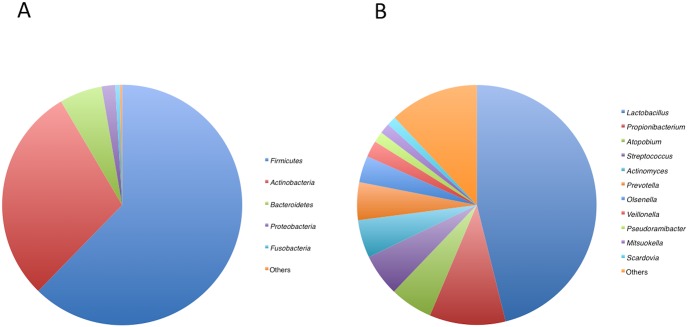
Mean bacterial distributions of bacterial phyla (A) and genera (B) in carious dentin.

The majority of the sequences were assigned to five bacterial phyla (Firmicutes, Actinobacteria, Bacteriodetes, Proteobacteria, and Fusobacteria; Figure 2A). Additionally, TM7, Cyanobacteria, and Spirochaetes were present in much lower proportions. The sequences represented 79 bacterial genera, and the mean relative abundances of 11 genera, such as *Lactobacillus* and *Propionibacterium*, exceeded 1% of the total reads (Figure 2B).

**Figure 3:**
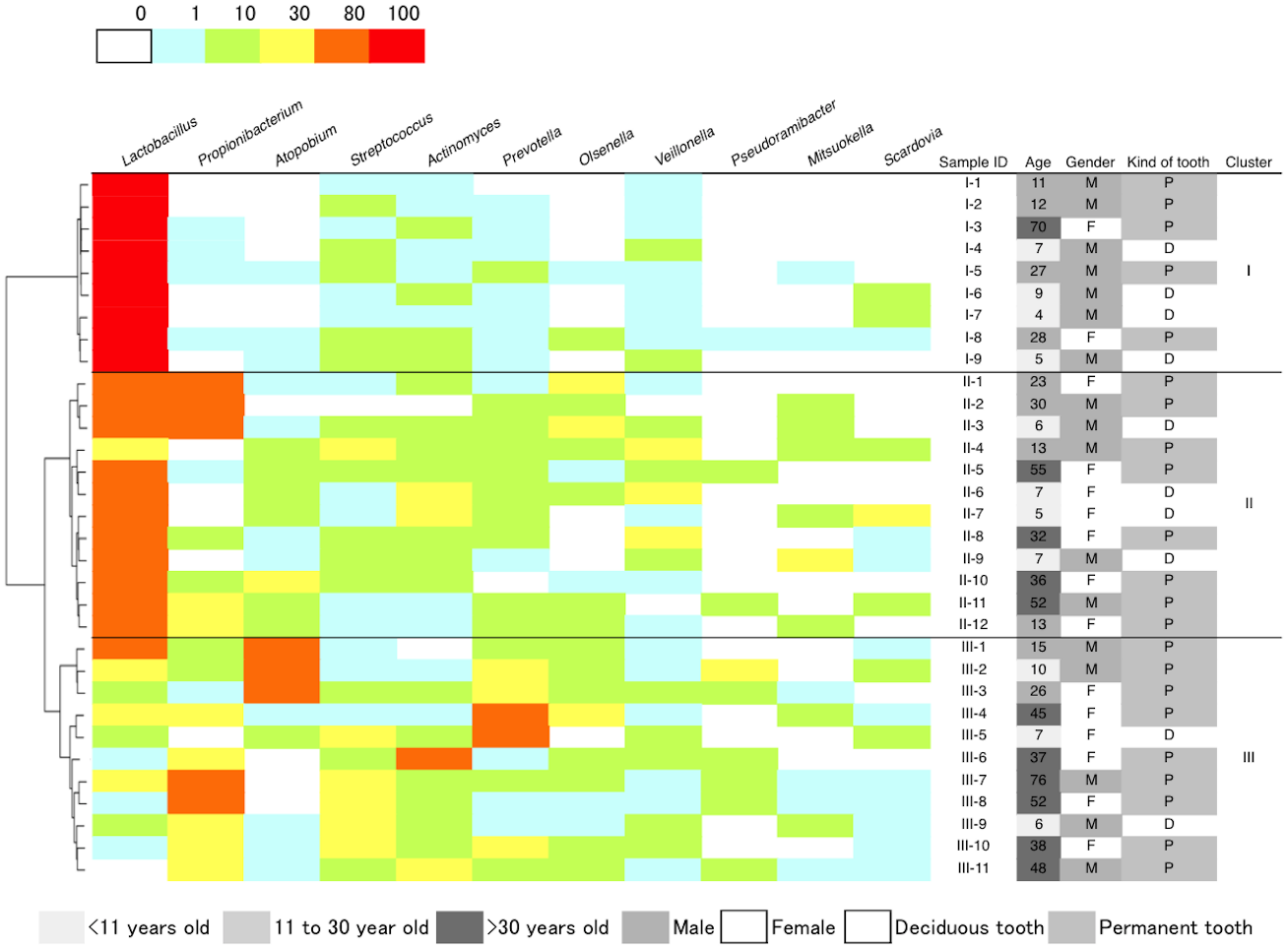
Relative abundance distribution of the bacterial genera in the 32 samples collected from the deepest layer of each region. Only 11 bacterial genera for which mean relative abundance exceeded 1% of total reads are described. Hierarchical cluster analysis using Euclidean distance and Ward’s method classified them into three clusters, according to the relative abundance of Lactobacillus: High-Lactobacillus group (cluster I), Mid-Lactobacillus group (cluster II) and Low-Lactobacillus group (cluster III). Age, gender of each subject, and deciduous or permanent tooth are indicated on the right side of the heatmap.

Because of the smaller variation in bacterial community composition among different depths in the same lesion compared with the inter-individual difference (Figure 1), the deepest sample was used to represent each lesion for which two or more samples were obtained. The relative abundance distribution of the 11 bacterial genera in the 32 samples collected from the deepest layer of each region is described in Figure 3. Hierarchical cluster analysis classified the bacterial community composition of genera into three clusters according to the relative abundance of *Lactobacillus*: high (cluster I), middle (cluster II), and low (cluster III). The bacterial composition pattern in cluster II was composed of high proportions of *Olsenella* and *Propionibacterium* or subdominated by heterogeneous genera. The bacterial communities in cluster III were dominated by *Atopobium*, *Prevotella*, or *Propionibacterium* with *Streptococcus* or *Actinomyces*. In the low-*Lactobacillus* group (cluster III), 6 of the 11 samples were collected from adults >30 years of age, while only 1 of the 9 samples in the high-*Lactobacillus* group (cluster I) were collected from adults >30 years of age (Figure 3).

A total of 25 out of 32 samples were further analyzed using the clone library method to confirm the results obtained by barcoded pyrosequencing with a short read length. The results obtained by the two methods were consistent with each other as described in Tables 2–5. The major species belonging to the genera *Atopobium* and *Propionibacterium*, which had not previously been identified as predominant bacterial genera in carious dentin, were *Atopobium* sp. HOT-416 and *Propionibacterium acidifaciens*, respectively (Tables 3–5). Furthermore, the major species belonging to the genus *Olsenella* were *Olsenella profusa* and *Olsenella* sp. HOT-807. The major species belonging to the genus *Streptococcus* were *S. mutans*, *Streptococcus oralis*, *Streptococcus salivarius*, *Streptococcus sanguinis*, and *Streptococcus vestibularis*. The *Lactobacillus* species identified in this study were consistent with those reported previously [10].

**Table 2:** Bacterial species identified in 3 carious dentin samples of Cluster I.

		Clone library analysis Number of clones			Pyrosequence analysis % of total reads		
Bacterial Species	Human Oral Taxon ID	I-2	I-3	I-9	I-2	I-3	I-9
*Lactobacillus*							
*L. acidophilus*	529						
*L. crispatus*	817		41			34.8	
*L. fermentum*	608	1		5			
*L. gasseri*	615	1	18	10	4.9	28.5	16.9
*L. oris*	709		1			2.6	
*L. paracasei*	716	1		3	3.1		5.2
*L. rhamnosus*	749						
*L. salivarius*	756			14		1.2	20.2
*L. vaginalis*	51		21			17.9	
*Lactobacillus.* sp	461	82			52.1		
*L. delbrueckii* a				9			
*L. fermentum* a				1			
*L. gallinarum* a			1				
*L. nagelli* a				1			
*L. reuteri* a		5					
*Lactobacillus* sp. str.RA2062^a^				4			
*Propionibacterium*							
*P. acidifaciens*	191					0.2	
*P. propionicum*	739					0.1	
*Atopobium*							
*A. parvilum*	723			1			0.1
*A. rimae*	750						
*Atopobium* sp.	416						
*Streptococcus*							
*S. mutans*	686			28		0.1	9.1
*S. oralis*	707						0.1
*S. salivarius*	755				0.6		
*S. sanguinis*	758				0.3		
*S. vestibularis*	21						
*Actinomyces*							
*A. gerencseriae*	618						
*Actinomyces* sp.	169					0.3	0.4
*Actinomyces* sp.	170						
*Actinomyces* sp.	171			1		0.1	
*Actinomyces* sp.	180						0.1
*Actinomyces* sp.	446						
*Actinomyces* sp.	448					0.1	0.8
*Actinomyces* sp.	449						
*Actinomyces* sp.	525						
*Prevotella*							
*P. denticola*	291						
*P. histicola*	298						0.3
*P. multisaccharivorax*	794						
*P. oris*	311						
*P. salivae*	307						
*P. veroralis*	572						
*Prevotella* sp	292						
*Prevotella sp*	300						
*Olsenella*							
*O. profusa*	806						
*Olsenella* sp.	807						
*Veillonella*							
*V. parvula*	161			1	0.5		2.7
*Veillonella. sp*	158						
*Pseudoramibacter*							
*P. alactolyticus*	538						
*P. alactolyticus* a							
*Mitsuokella*							
*Mitsuokella* sp.	131						
*Scardovia*							
*S. inopinata*	642						
*S. wiggsiae*	195						
Others					38.5	14.1	44.1
Total		90	82	78	100	100	100

a Sequences with no hits (<98% identity) in Human Oral Microbiome Database were further compared against database Nucleotide collection database (nr/nt) of the National Center for Biotechnology Information.

**Table 3:** Bacterial species identified in 8 carious dentin samples belonging to Cluster II.

		Clone library analysis Number of clones								Pyrosequence analysis % of total reads							
Bacterial Species	Human Oral Taxon ID	II-1	II-3	II-4	II-6	II-7	II- 10	II- 11	II- 12	II-1	II-3	II-4	II-6	II-7	II- 10	II- 11	II- 12
*Lactobacillus*																	
*L. acidophilus*	529						3										
*L. crispatus*	817				3			8					4.3			2.0	
*L. fermentum*	608																
*L. gasseri*	615				28	45	14	2	9	8.2	0.1		14.2	37.2	29.0	0.5	18.2
*L. oris*	709						6								10.0		
*L. paracasei*	716			21	18		1	7	19	30.6		16.4	20.9	1.9	1.6		26.8
*L. rhamnosus*	749		1					2			17.0			0.5		25.2	
*L. salivarius*	756							7	11							22.6	13.6
*L. vaginalis*	51							4	1						0.3	1.3	0.1
*Lactobacillus.* sp	461																
*L. delbrueckii* a			13														
*L. fermentum* a																	
*L. gallinarum* a																	
*L. nagelli* a																	
*L. reuteri* a																	
*Lactobacillus* sp. str.RA2062^a^																	
*Propionibacterium*																	
*P. acidifaciens*	191	11	21					11	4	19.3	29.3				0.3	11.2	13.1
*P. propionicum*	739														0.8		
*Atopobium*																	
*A. parvilum*	723									0.1		1.7	0.4	0.3			0.3
*A. rimae*	750			2	10					0.1		5.1	6.4	0.5			0.6
*Atopobium* sp.	416						66	6			0.2				19.4	4.3	
*Streptococcus*																	
*S. mutans*	686		1							0.2	1.3				1.2		
*S. oralis*	707																
*S. salivarius*	755			2						0.2		8.1					
*S. sanguinis*	758											0.7					
*S. vestibularis*	21			4								0.1					
*Actinomyces*																	
*A. gerencseriae*	618						1					0.1	0.4	1.2	3.2		
*Actinomyces* sp.	169									0.8		0.3		0.1			
*Actinomyces* sp.	170										0.2			0.1	0.1	0.1	
*Actinomyces* sp.	171			4	4	1						0.1		0.1		0.1	
*Actinomyces* sp.	180											0.1			0.1		
*Actinomyces* sp.	446																
*Actinomyces* sp.	448													0.4	1.0	0.1	
*Actinomyces* sp.	449									0.1			0.8				
*Actinomyces* sp.	525																
*Prevotella*																	
*P. denticola*	291			1							1.0	3.3	1.4				1.2
*P. histicola*	298									0.1		0.1	0.2				0.1
*P. multisaccharivorax*	794															3.9	
*P. oris*	311									0.2	0.2			0.1			
*P. salivae*	307										0.2	0.1	0.2	0.4			
*P. veroralis*	572									0.1	1.0	0.4	0.2	4.3			
*Prevotella* sp	292					1					0.3			1.2			
*Prevotella sp*	300					1											
*Olsenella*																	
*O. profusa*	806	73	51					17	1	23.7	9.8					7.5	0.9
*Olsenella* sp.	807				3							2.9	6.5				
*Veillonella*																	
*V. parvula*	161				2					0.2	1.3	9.6	14.6	0.2	0.5		
*Veillonella. sp*	158				5												
*Pseudoramibacter*																	
*P. alactolyticus*	538							3									
*P. alactolyticus* a								6									
*Mitsuokella*																	
*Mitsuokella* sp.	131			11		1					3.0	9.6		6.3			5.2
*Scardovia*																	
*S. inopinata*	642							18								1.3	
*S. wiggsiae*	195			38		39						6.0		12.8			
Others		7	3	8	6		1	1	50	16.1	35.1	35.3	29.5	32.4	32.5	19.9	19.9
Total		91	90	91	79	88	92	92	95	100	100	100	100	100	100	100	100

a Sequences with no hits (<98% identity) in Human Oral Microbiome Database were further compared against database Nucleotide collection database (nr/nt) of the National Center for Biotechnology Information.

**Table 4:** Bacterial species identified in 5 carious dentin samples belonging to Cluster III (III-1–5).

		Clone library analysis Number of clones					Pyrosequence analysis % of total reads				
Bacterial Species	Human Oral Taxon ID	III-1	III-2	III-3	III-4	III-5	III-1	III-2	III-3	III-4	III-5
*Lactobacillus*											
*L. acidophilus*	529										
*L. crispatus*	817										
*L. fermentum*	608										
*L. gasseri*	615							0.1	13.4	1.7	0.1
*L. oris*	709						2.2				
*L. paracasei*	716			1		1			11.8	1.8	2.6
*L. rhamnosus*	749								0.8	1.3	
*L. salivarius*	756							0.2			
*L. vaginalis*	51						3.7		4.4		
*Lactobacillus.* sp	461										
*L. delbrueckii* a											
*L. fermentum* a											
*L. gallinarum* a											
*L. nagelli* a											
*L. reuteri* a											
*Lactobacillus* sp. str.RA2062^a^											
*Propionibacterium*											
*P. acidifaciens*	191						0.5	3.7		10.2	
*P. propionicum*	739										
*Atopobium*											
*A. parvilum*	723					1					0.2
*A. rimae*	750					3				0.4	3.4
*Atopobium* sp.	416	92	95	91			52.1	30.6	48.1		
*Streptococcus*											
*S. mutans*	686					30	0.2			0.3	24.4
*S. oralis*	707									0.1	0.1
*S. salivarius*	755									0.1	0.1
*S. sanguinis*	758						0.1				
*S. vestibularis*	21										
*Actinomyces*											
*A. gerencseriae*	618										
*Actinomyces* sp.	169						0.1				0.1
*Actinomyces* sp.	170										0.1
*Actinomyces* sp.	171										
*Actinomyces* sp.	180						0.4				0.1
*Actinomyces* sp.	446										
*Actinomyces* sp.	448					1	0.1	0.1		0.1	4.3
*Actinomyces* sp.	449										
*Actinomyces* sp.	525										
*Prevotella*											
*P. denticola*	291						0.1		1.2	0.5	
*P. histicola*	298					1			0.3		7.9
*P. multisaccharivorax*	794				21			7.6		31.0	
*P. oris*	311					1				0.1	0.9
*P. salivae*	307					1		0.1			1.0
*P. veroralis*	572					4		0.1	0.1	1.4	3.4
*Prevotella* sp	292										
*Prevotella sp*	300										
*Olsenella*											
*O. profusa*	806		1		70		1.2	3.7	0.1	18.7	
*Olsenella* sp.	807										
*Veillonella*											
*V. parvula*	161						1.0	0.1	0.2	0.1	1.9
*Veillonella. sp*	158										
*Pseudoramibacter*											
*P. alactolyticus*	538						2.3				
*P. alactolyticus* a											
*Mitsuokella*											
*Mitsuokella* sp.	131						0.1			2.1	
*Scardovia*											
*S. inopinata*	642							2.1			
*S. wiggsiae*	195					41			0.1	0.2	5.2
Others					1	1	35.9	51.6	19.5	29.9	44.2
Total		92	96	92	92	85	100	100	100	100	100

a Sequences with no hits (<98% identity) in Human Oral Microbiome Database were further compared against database Nucleotide collection database (nr/nt) of the National Center for Biotechnology Information.

**Table 5:** Bacterial species identified in 6 carious dentin samples belonging to Cluster III (III-6–11).

		Clone library analysis Number of clones						Pyrosequence analysis % of total reads					
Bacterial Species	Human Oral Taxon ID	III-6	III-7	III-8	III-9	III- 10	III- 11	III-6	III-7	III-8	III-9	III- 10	III- 11
*Lactobacillus*													
*L. acidophilus*	529												
*L. crispatus*	817											0.1	
*L. fermentum*	608		1										
*L. gasseri*	615	3							0.7		0.4		
*L. oris*	709												
*L. paracasei*	716										0.6	0.5	
*L. rhamnosus*	749												
*L. salivarius*	756		1						3.1				
*L. vaginalis*	51							0.3					
*Lactobacillus.* sp	461												
*L. delbrueckii* a													
*L. fermentum* a													
*L. gallinarum* a													
*L. nagelli* a													
*L. reuteri* a													
*Lactobacillus* sp. str.RA2062^a^													
*Propionibacterium*													
*P. acidifaciens*	191	25	39	58	22	65	35	21.0	48.3	46.0	19.5	28.2	16.8
*P. propionicum*	739						3						1.6
*Atopobium*													
*A. parvilum*	723												
*A. rimae*	750										0.8		0.1
*Atopobium* sp.	416					2					0.1	0.1	0.2
*Streptococcus*													
*S. mutans*	686		11	9	56			0.8	20.6	14.1	25.6	0.8	
*S. oralis*	707						1				0.1		0.7
*S. salivarius*	755			7						0.4	0.1	0.1	0.1
*S. sanguinis*	758						2	0.4		2.8	1.1	0.1	1.6
*S. vestibularis*	21												
*Actinomyces*													
*A. gerencseriae*	618							0.2			1.3	0.1	
*Actinomyces* sp.	169	42						43.1	0.4		0.2	0.2	
*Actinomyces* sp.	170		1					0.3		0.2		0.2	0.2
*Actinomyces* sp.	171	2				1							
*Actinomyces* sp.	180						1		0.1			0.1	0.1
*Actinomyces* sp.	446			3									
*Actinomyces* sp.	448	3				1		4.1	0.1	3.7	0.8	2.2	0.6
*Actinomyces* sp.	449			1			5			0.1			5.3
*Actinomyces* sp.	525						3						0.1
*Prevotella*													
*P. denticola*	291					3			0.1	0.1	0.2	14.9	0.4
*P. histicola*	298								0.1			0.1	
*P. multisaccharivorax*	794							0.2	1.2				2.7
*P. oris*	311											0.3	
*P. salivae*	307												
*P. veroralis*	572										0.1	1.1	0.4
*Prevotella* sp	292											0.6	
*Prevotella sp*	300												
*Olsenella*													
*O. profusa*	806	5	27			17	9	4.5	6.6	3.2	0.1	5.1	4.2
*Olsenella* sp.	807						1						0.8
*Veillonella*													
*V. parvula*	161	1						1.1			3.7	1.6	0.4
*Veillonella. sp*	158												
*Pseudoramibacter*													
*P. alactolyticus*	538							1.2	1.6	6.3			
*P. alactolyticus* a							2						
*Mitsuokella*													
*Mitsuokella* sp.	131			1					0.2	0.1	4.8		0.2
*Scardovia*													
*S. inopinata*	642								0.5			0.3	
*S. wiggsiae*	195			4						0.1	0.2		0.1
Others		8	5	10	11	2	25	22.8	16.4	22.9	40.3	43.3	63.4
Total		89	85	93	89	91	87	100	100	100	100	100	100

a Sequences with no hits (<98% identity) in Human Oral Microbiome Database were further compared against database Nucleotide collection database (nr/nt) of the National Center for Biotechnology Information.

## Discussion

In the present study, we analyzed the composition of the microbiota in carious dentine lesions utilizing next-generation sequencing. Prior to BLAST analysis using the HOMD, all of the sequences were directlyclustered into OTUs using the RDP pyrosequencing pipeline, and representative sequences of each cluster were selected using the Dereplicate request function. Hierarchical cluster analysis of the genera obtained by this method resulted in three main clusters based on the relative abundance of 10 major genera having >1% occupancy as shown in Figure S1; notably, the results are similar to those in Fig. 3. However, *Atopobium* was predominant in cluster III using the clone library method, but it was not identified as a major genus in Figure S1. *Atopobium* was proposed as a new genus derived from *Lactobacillus* and *Streptococcus* with the type strain *Atopobium minutum* in 1992 based on comparative sequence analyses [26]. A close relationship between *Atopobium* and *Olsenella* was shown in the bigeneric *Olsenella*-*Atopobium* branch of the family Coriobacteriaceae, class Actinobacteria [27], which might have caused misidentification in our first RDP pyrosequencing pipeline attempt. We confirmed that the RDP Classifier misclassified the V1–V2 region of the 16S rRNA gene of *Atopobium* sp. HOT-416 as *Olsenella*, resulting in erroneous pyrosequencing results due to the short read length. Furthermore, appropriate assignment of the sequences produced by pyrosequencing to bacterial species using the BLAST method and the HOMD was confirmed by the clone library method as shown in Tables 2–5. Considering these facts, pyrosequencing with the BLAST method and the HOMD is thought to be superior to the RDP pipeline method for evaluating the microbiota of dentin caries. Therefore, we chose the BLAST assignment method using the HOMD for the analysis of our pyrosequencing data. In addition, a considerable discrepancy was found in species occupancy between the pyrosequencing and clone library analyses. A recent study confirmed the superior quantitative accuracy of pyrosequencing compared with clone library analysis [28], suggesting that pyrosequencing analysis utilizing the BLAST assignment method and the HOMD is a promising systemic evaluation method for determining the microbiota in carious dentine lesions.

We comprehensively analyzed the bacterial compositions of 44 dentinal caries samples from 32 Japanese dental patients with a broad age range (4–76 years) using pyrosequencing analysis with the BLAST assignment method and the HOMD to provide a general characterization of this composition in the Japanese population. Then, we sought to isolate the deepest area of each dentinal caries lesion after removing the surface area contaminated with saliva. Nine thick lesions were divided into two or three samples each according to depth. However, PCoA analysis of sequences from 44 samples suggested that the bacterial compositions in samples from the same lesion were similar. This result is consistent with that of Munson et al. [9], suggesting that variation in bacterial composition seems to extend beyond depth-related differences in the same dentinal caries lesion when saliva-contaminated portions have been appropriately eliminated. Thus, we concentrated on individual-level variation in bacterial composition using samples from the deepest area of each lesion, which is the region of dentinal caries progression.

Eleven genera (*Lactobacillus, Propionibacterium, Atopobium, Streptococcus, Prevotella, Olsenella, Actinomyces, Veillonella, Pseudoramibacter, Mitsuokella*, and *Scardovia*) were the major members, each occupying >1% of the total reads. Relatively low-diversity compositions were observed compared with those in saliva and dental plaque, suggesting that environmental pressures within a dentinal caries lesion, such as low pH and limited nutrients, might select bacterial species that can survive in such conditions. Whether all of these species are indispensable for dentinal caries progression remains to be elucidated.

Cluster analysis revealed that *Lactobacillus* dominated one-third of all samples (cluster I), as in dentinal caries cases previously characterized by culturing and molecular methods [9], [10], [29]. The great ability of *Lactobacillus* species to produce organic acids suggests that these species are responsible for dentinal destruction. On the other hand, the ability of *Lactobacillus* to destroy organic substances, especially collagen, has not been characterized. Host matrix metalloproteinases (MMPs) have recently been suggested to play an important role in the degradation of demineralized dentin organic matrix, with activation by bacterial acids [30]. MMPs present in saliva (MMP-1 and -8) and sound dentin (MMP-1, -2, -3, -9, and -20) are promising candidates for the degradation of demineralized dentin organic matrix [31], and host susceptibility to dental caries might depend on variation in MMP activity in saliva and dentin. Dentin organic matrix destruction associated with the bacterial composition of cluster I is likely attributable to host proteinases, not bacteria. The bacterial compositions of several samples in cluster II (II-4–12) were similar to those in cluster I, with minor differences in the quantities of some genera generally found in saliva or dental plaque. Although the analysis distinguished these samples from those in cluster I, these samples seemed to be a subclass of cluster I. In addition, we could not exclude the possibility that minor genera in these samples were derived from contamination by saliva or plaque, but the failure to recognize >1% *Neisseria* in all sequences suggests that the probability of such contamination was low. As previously shown, *Lactobacillus* species might play an important role in dentinal caries progression [9], [10], [29], in collaboration with other minor genera found in this cluster.

In carious dentine lesions, various species of *Lactobacillus* have been detected at high frequencies and in large quantities, and most cases lacking mutans streptococci were among British and Australian populations [9] [10], suggesting that mutans streptococci are not necessarily required for the progression of dentinal caries. In the present study, mutans streptococci were detected in only about one-fourth of samples subjected to clone library analysis, and large populations were found in only two samples. It is likely that mutans streptococci do not play an important role in dentin caries progression in Japanese populations.

*Prevotella* is another predominant genus in dentinal caries [10]–[12]. In the present study, the previously characterized *Prevotella-*predominant type was only found in two samples in cluster III. However, this type was detected in <10% of subjects and likely has a minor prevalence in Japanese populations, perhaps due to the typical carbohydrate-rich diet. Although the significance of *Prevotella* in dentinal caries has not been determined, these bacteria may produce proteolytic enzymes responsible for the degradation of dentin organic matrix, as many *Prevotella* species exhibit strong proteolytic activity [32], although host MMP activity should not be ignored.

Some samples with predominant *Atopobium* or *Propionibacterium* in clusters II and III were identified as novel combinations of microbiota in dentin caries lesions and might be characteristic of Japanese populations. *Atopobium* was predominant in three samples in cluster III, and some contribution of *Atopobium* sp. HOT-416 to dentinal caries development was proposed [5], [33]. These previous reports are consistent with our finding that almost all sequences of *Atopobium* were *Atopobium* sp. HOT-416. However, no previous reports have described a dentinal caries microbiota dominated by *Atopobium*, and this is the first report of an *Atopobium*-predominant microbiota related to dentinal caries; again, this type may be characteristic of Japanese populations. *Propionibacterium acidifaciens* was found in clusters II and III, and was dominant in half of the samples in cluster III. Although *P. acidifaciens* has been associated with caries in young permanent teeth [34] and root caries in elderly patients [35], the role of this bacterium in dentin caries lesions has not been defined. This species coexisted with high levels of *S. mutans* or *Actinomyces* sp. HOT-169 in relatively heterogeneous microbiota, which may be important for the ability of *P. acidifaciens* to cause dentinal caries progression. *P. acidifaciens*-predominant microbiota in cluster III, which occupied one-fifth of all samples and one-half of the samples that were low in *Lactobacillus*, may be important in the etiology of dentinal caries in the Japanese population.

It is more ideal to explore the association between composition of microbiota of dental caries lesion and that of dental plaque on healthy tooth surfaces within the same individual. However, the definition of plaque on a healthy surface is complex. It is not easy to decide which tooth and which surface are appropriate for plaque sampling and what timing (after or before brushing, after or before meal) is appropriate for plaque sampling. Controlling the timing of plaque sampling in clinical patients is difficult, and designing such a study would be problematic. Exploring the association between the composition of the microbiota of dental caries lesions and that of dental plaque on healthy tooth surfaces is beyond the scope of the present study. We aim to address this matter in a future study.

In this study, sham extractions as a control were not included in the 16S rRNA gene amplification procedure. Therefore, we could not exclude the possibility that our results contain contaminated bacterial species. Careful consideration would be needed to identify minor bacterial species observed in our study as members of carious dentin microbial community.

In the present study, we summarized the bacterial compositions of dentinal caries lesions in a Japanese population using next-generation sequencing and found typical Japanese types: *Atopobium*- or *Propionibacterium*-predominant microbiota. On the other hand, we were surprised to find that the components of these clusters (*Atopobium* sp. HOT-416 and *P. acidifaciens*) were the same at the species level as those identified in other geographic regions [5], [9], [33]–[35]. Furthermore, *Atopobium* sp. HOT-416 and *P. acidifaciens* may play important roles in dentinal caries lesions with low quantities of *Lactobacillus* species. The properties of these species and the combination of *Atopobium* sp. HOT-416 with *S. mutans* or *Actinomyces* sp. HOT-169 are future targets in understanding dentinal caries etiology.

## Supporting Information

Figure S1 Relative abundance distribution of the bacterial genera in the 32 samples collected from the deepest layer of each region analyzed by using the RDP pyrosequencing pipeline. Only 10 bacterial genera for which mean relative abundance exceeded 1% of total reads are described. Hierarchical cluster analysis using Euclidean distance and Ward’s method classified them into three clusters, according to the relative abundance of Lactobacillus: High-Lactobacillus group (cluster I), Mid-Lactobacillus group (cluster II) and Low-Lactobacillus group (cluster III). Age, gender of each subject, and deciduous or permanent tooth are indicated on the right side of the heatmap.
(TIFF)
