# Supplementary figures and images for: Identification of the Microbiota in Carious Dentin Lesions Using 16S rRNA Gene Sequencing
Source: PLoS One. 2014 Aug 1;9(8):e103712. doi: 10.1371/journal.pone.0103712 (PMC4118920; doi:10.1371/journal.pone.0103712)

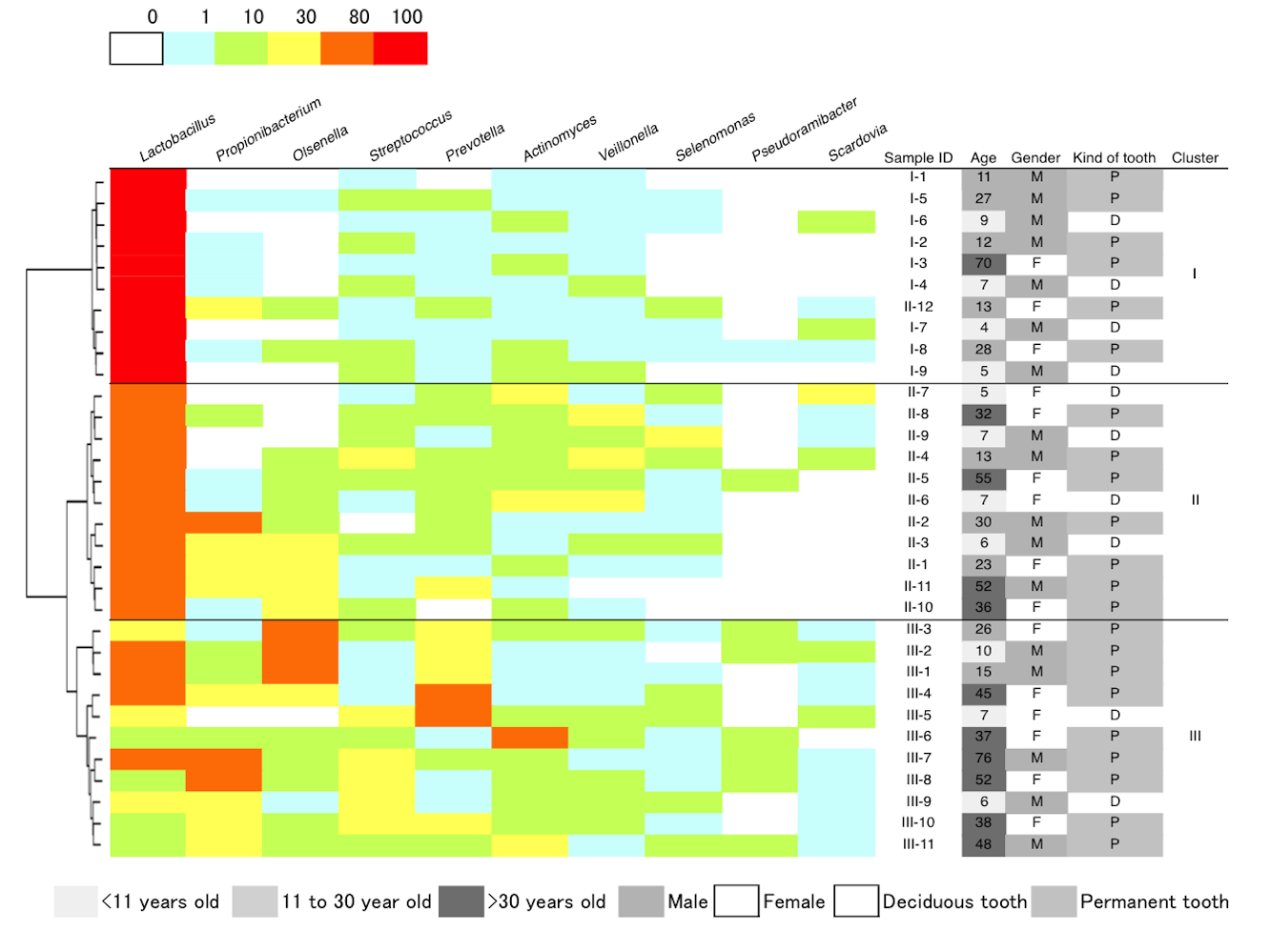

Supplement: Figure S1 — Relative abundance distribution of the bacterial genera in the 32 samples collected from the deepest layer of each region analyzed by using the RDP pyrosequencing pipeline. Only 10 bacterial genera for which mean relative abundance exceeded 1% of total reads are described. Hierarchical cluster analysis using Euclidean distance and Ward’s method classified them into three clusters, according to the relative abundance of Lactobacillus: High-Lactobacillus group (cluster I), Mid-Lactobacillus group (cluster II) and Low-Lactobacillus group (cluster III). Age, gender of each subject, and deciduous or permanent tooth are indicated on the right side of the heatmap. (TIFF) [file pone.0103712.s001.tif]
